# A population-level computational histologic signature for invasive breast cancer prognosis

**DOI:** 10.21203/rs.3.rs-2947001/v1

**Published:** 2023-05-26

**Authors:** Mohamed Amgad, James Hodge, Maha Elsebaie, Clara Bodelon, Samantha Puvanesarajah, David Gutman, Kalliopi Siziopikou, Jeffery Goldstein, Mia Gaudet, Lauren Teras, Lee Cooper

**Affiliations:** Department of Pathology, Northwestern University, Chicago, IL, USA; American Cancer Society; Department of Medicine, Cook County Hospital, Chicago, IL, USA; Department of Population Science, American Cancer Society; Department of Population Science, American Cancer Society; Department of Biomedical Informatics, Emory University School of Medicine, Atlanta, Georgia, USA; Department of Pathology, Northwestern University Feinberg School of Medicine; Northwestern University, Feinberg School of Medicine; Division of Cancer Epidemiology and Genetics, National Cancer Institute; Department of Population Science, American Cancer Society; Northwestern University

## Abstract

Breast cancer is a heterogeneous disease with variable survival outcomes. Pathologists grade the microscopic appearance of breast tissue using the Nottingham criteria, which is qualitative and does not account for non-cancerous elements within the tumor microenvironment (TME). We present the Histomic Prognostic Signature (HiPS), a comprehensive, interpretable scoring of the survival risk incurred by breast TME morphology. HiPS uses deep learning to accurately map cellular and tissue structures in order to measure epithelial, stromal, immune, and spatial interaction features. It was developed using a population-level cohort from the Cancer Prevention Study (CPS)-II and validated using data from three independent cohorts, including the PLCO trial, CPS-3, and The Cancer Genome Atlas. HiPS consistently outperformed pathologists’ performance in predicting survival outcomes, independent of TNM stage and pertinent variables. This was largely driven by stromal and immune features. In conclusion, HiPS is a robustly validated biomarker to support pathologists and improve prognosis.

## Introduction

**Figure 1:**
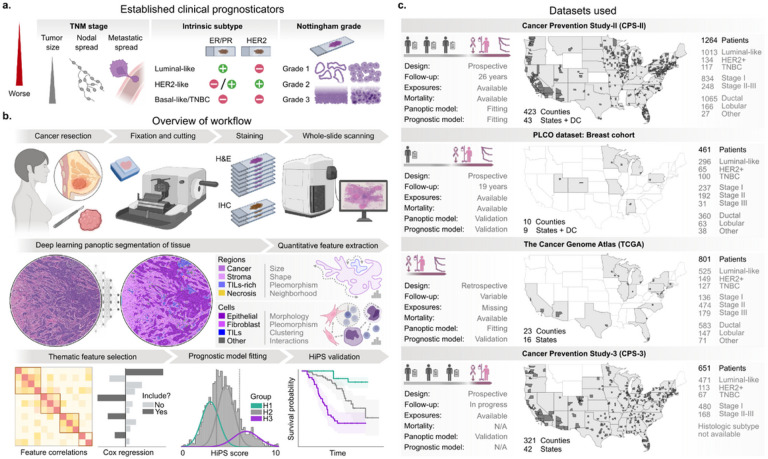
Overview of the methodological approach and datasets used. a. Established clinical prognosticators in breast cancer. The AJCC Staging Manual defines three treatment-oriented subtypes: Luminal-like cancer patients are eligible for hormone therapy, HER2-like cancer patients are eligible for Trastuzumab, and TNBC patients are not eligible for targeted therapies. We limited our analysis to invasive cancers, not metastatic at the time of diagnosis. All specimens are routinely assessed using the standard IHC/ISH panel (ER/PR/HER2) and H&E-stained slides. b. Our workflow for determining the Histomic Prognostic Signature (HiPS). Breast cancer resection specimens were fixed in formalin, embedded in paraffin, cut, stained, and digitally scanned. A panoptic segmentation model identified tissue regions and nuclei in each slide, followed by the computational extraction of interpretable morphologic features. These features include stromal, immune, and spatial interaction features not included in Nottingham grading. The most prognostic features within each biologic theme, combined with ER/PR/HER2, were used to fit a Cox regression model to cancer-specific survival data. The resultant HiPS score is an interpretable weighted combination of histologic features. Additionally, we learned thresholds to identify three distinct prognostic groups. Finally, we validated HiPS using clinical, genomic, and epidemiologic data. c. An overview of the datasets used. We include patients from almost all geographic regions from the United States, covering 614 counties in 48 states, plus the District of Columbia. CPS-II data was used for prognostic model fitting, while PLCO, TCGA, and CPS-3 were independent validation cohorts. Prediagnostic risk factor exposure data were available for all datasets except TCGA, while survival outcomes were available for all datasets except CPS-3. TCGA and PLCO specimens were exclusively sourced from tertiary medical centers, unlike the CPS datasets, which were mostly sourced from non-tertiary and community hospitals. Partially created with BioRender.com.

Breast cancer is the most common malignancy worldwide^1,2^. It is a heterogeneous disease with highly variable survival outcomes that depend on tumor biology, therapeutic regimen, and socioeconomic determinants of health^3,4^. Established prognostic criteria include the American Joint Committee on Cancer (AJCC) tumor-node-metastasis (TNM) staging, Nottingham histologic grading, and intrinsic subtype. Intrinsic subtype can be determined by gene expression profiling or approximated using immunohistochemistry (IHC) or in-situ hybridization (ISH)-based assessment of the Estrogen (ER), Progesterone (PR), or HER2 receptor expression (Fig. 1a). Starting 2018, the AJCC manual introduced the *prognostic stage*, which combines the traditional TNM stage with ER/PR/HER2 status and Nottingham grade^3^. This shift in consensus reflects the necessity and complexity of combining multimodal information to place patients along a risk spectrum^5^.

In this paper, we present an artificial intelligence system to improve the prognostication of patients with non-metastatic invasive carcinomas of the breast. Consistent with AJCC staging, we devised a *Histomic Prognostic Signature* (HiPS) risk score that combines modalities available to every pathologist: Hematoxylin and Eosin (H&E)-stained slides and the ER/PR/HER2 panel. HiPS addresses three issues with the current standard of care. First, we use advanced deep learning-based computer vision for quantitative assessment of whole-slide images (WSI) of slides, providing an objective alternative that mitigates variability inherent in manual Nottingham grading, and captures latent features that cannot be reliably graded.

Second, we comprehensively assess the entire tumor microenvironment (TME) including non-neoplastic elements (Figs. S1–5). The Nottingham grading criteria, which assign patients into grades G1-G3, was standardized in the 1930s-1990s^6–10^. In the decades since, it has become clear that cancer progression involves multiple pathologic processes, including cancer-permissive inflammation, activation of wound healing and repair cascades, immune modulation, hypoxia-driven metabolic derangements, epithelial-to-mesenchymal transition, and spatio-geometric changes that enable the invasion of cancer cells^11^. Many of these hallmarks are reflected in changes to the density, appearance, and spatial clustering of cancer-associated fibroblasts (CAFs), tumor-infiltrating lymphocytes (TILs), and stromal matrix^12–19^. HiPS measures these morphologic changes using a comprehensive, interpretable set of features not captured by visual grading.

Third, we combine the standard ER/PR/HER2 panel with histologic features in a unified framework. This integration enables us to accurately gauge the prognostic importance of the TME morphology while accounting for the effect of intrinsic subtype. Indeed, several recent studies showed that tumor morphology differs with hormone receptor expression^20–23^.

HiPS was developed using a large prospective cohort from the American Cancer Society’s (ACS) Cancer Prevention Study-II (CPS-II). Cancer-free participants were enrolled from the general population, unselected for specific characteristics^24^. We validated HiPS using three independent cohorts, including the Prostate Lung Colorectal and Ovarian cancer screening trial (PLCO), The Cancer Genome Atlas (TCGA), and the ACS’s Cancer Prevention Study-3 (CPS-3)^25–27^. Collectively, the cases were diagnosed in hundreds of US healthcare facilities, ranging from large academic cancer centers to rural facilities (Fig. 1c, Tables S1–4). This diversity supports the broad applicability of HiPS as a generalizable biomarker.

## Results

### Panoptic segmentation enables extraction of interpretable TME biomarkers

Before the widespread adoption of deep learning, automated grading workflows relied on detecting tissue regions and nuclei based on shape, texture, and contextual heuristics^28–30^. This approach was largely replaced by deep learning methods that learn features of relevance to the final prediction^31–33^. By predicting survival directly from image pixels, bypassing cancer detection, these end-to-end approaches improved prediction accuracy^34–39^. However, this accuracy can come at the cost of explainability. Explainability techniques have been developed to address this shortcoming, including saliency heatmaps, LIME, and DTALE^40–42^. However, these approaches offer post hoc explanations and are vulnerable to confirmation bias^43,44^. Moreover, heatmap-based explanations offer little insight into the directionality or proportionality of influence on the model’s prediction.

**Figure 2:**
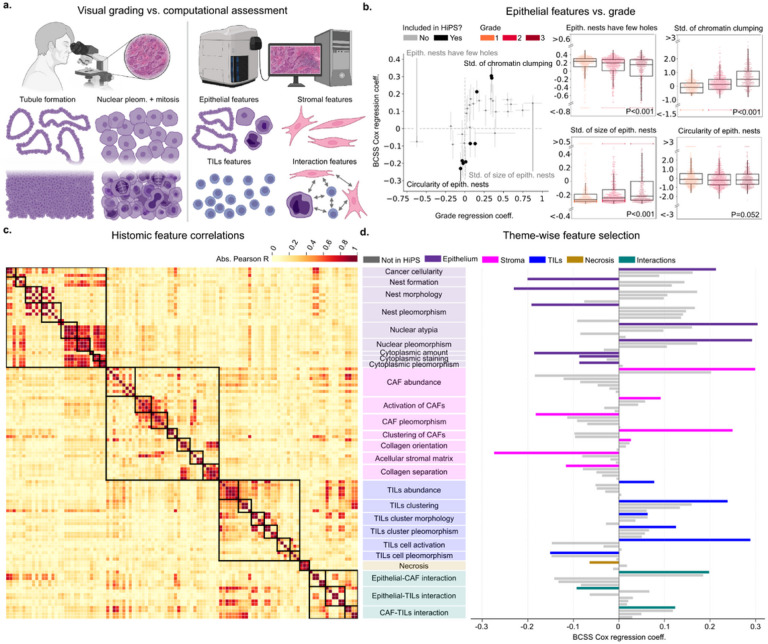
Thematic categorization and selection of features using the CPS-II cohort. Supporting results are provided in Tables S23-25. a. Conceptual differences between visual grading and computational assessment. Pathologists use the Nottingham grading criteria, a visual semi-quantitative aggregate score of epithelial tubule formation, nuclear pleomorphism, and mitotic figures. In contrast, our models quantitatively assess the entire tumor microenvironment, including stromal and immune cells, stromal matrix, and spatial interactions.b. Left: Association of epithelial histomic features with visual grading (ordinal regression) versus association with survival (Cox regression). Error bars represent the standard error. Right: Box plots of the feature value distributions of two histomic features highly associated with grade (left), and two highly associated with survival (right). Feature selection for HiPS was guided by the association with survival, not grade. In fact, the standard deviation of epithelial nest size closely captures grades, yet is only modestly prognostic compared to alternative epithelial features. P-values were obtained using the Kruskal-Wallis test. c.Inter-feature correlations. The squares represent biological themes and subthemes. Except for interaction features, cross-theme correlations are mostly weak, reflecting the independence of different themes. d. Univariable Cox regression coefficients for all 109 histomic features. The most prognostic feature within each of the 26 subthemes was included in the HiPS signature. Partially created with BioRender.com

To address these limitations, we used a *concept bottleneck modeling* approach to discover visual prognostic biomarkers of the TME (Fig. 1b)^45^. First, we used a multi-resolution panoptic segmentation model called *MuTILs* to delineate and classify tissue regions and cell nuclei in WSIs (Figs. S2–5)^46,47^. Second, we designed a comprehensive set of 109 features from 26 biological themes that describe the size, shape, texture, and contextual relationship of regions and nuclei; these are referred to as *histomic features* (Fig. 2, Table S5). Using univariable Cox regression of breast cancer-specific survival (BCSS), we identified the most prognostic feature from each theme (Table S23). The 26 most prognostic histomic features, along with the ER/PR/HER2 expression status, form the *Histomic Prognostic Signature* (HiPS). Finally, we use HiPS features in a regularized Cox proportional hazards model to obtain a continuous risk score, the *HiPS score*, in the range [0, 10]. Using Gaussian Mixture modeling of the score distribution, we identified thresholds to define *HiPS groups H1-H3*. Cancers scoring <3.6 are H1, while those scoring ≥6 are H3. For comparison, we fit a control model that combines the Nottingham grade with the ER/PR/HER2 expression status. Of note, the granularity of staging data we had precluded using the AJCC prognostic stage as our control. We refer to continuous and discrete predictions from the Control model as the *Control score* and *Control groups C1-C3* (Fig. S7).

Panoptic segmentation was trained using annotations from the Breast Cancer Semantic Segmentation and NuCLS datasets derived from 125 slides from the TCGA cohort, along with 85 annotated slides from the CPS-II cohort^24,25,46,48,49^. Slide visualization and management utilized the Digital Slide Archive platform^50^. For survival modeling, the CPS-II dataset was used for feature selection and to learn feature weights and score thresholds for HiPS and Control scoring/grading. Hence, CPS-II is our discovery cohort, TCGA is a semi-independent validation cohort (used for fitting panoptic segmentation, but not the survival model), and PLCO and CPS-3 are independent validation cohorts. Because TCGA and PLCO were selected for particular tumor characteristics and sourced exclusively from tertiary care centers, they differ from our discovery cohort. Specifically, there are significant differences in the clinical characteristics (Tables S1–4) as well as the distributions of HiPS features in these datasets (Figs. S25–29). Yet, HiPS performance was robust and consistent across cohorts.

### Epithelial features not captured by the Nottingham grade are highly prognostic

Many of the epithelial histomic features tested correspond to the Nottingham criteria. We studied this relationship by examining the strength of features’ association with grade versus their association with survival. Histomic features with the strongest Nottingham grade association were not necessarily the most prognostic (Figs. 2b,S19). In the CPS-II and CPS-3 cohorts, features capturing glandular architecture had the highest association with grading, including the mean and variance of epithelial nest area and perimeter, and the number of holes within epithelial nests and its variance (i.e., formation of a glandular lumen). In CPS-II we found that these features were less prognostic than other architectural features like the circularity of epithelial nests and epithelial cell clustering within a 64 μM radius. In fact, the most prognostic epithelial features capture nuclear morphology, namely chromatin clumping of epithelial nuclei (BCSS HR=1.35, p=0.001) and its variance (BCSS HR=1.34, p=0.001).

Nuclear features had the highest association with Nottingham grading in PLCO and TCGA (Fig S19). These include the size of epithelial nuclei, complexity of the epithelial nuclear boundary, and epithelial nuclear chromatin clumping. In PLCO, these features were also the most prognostic (BCSS HR range=1.72–3.61), and there was a direct relationship between how prognostic a histomic feature was and how well it was associated with the Nottingham grade. TCGA, on the other hand, had many top prognostic features that were not as strongly associated with grade (Figs. S23–24), including the number of “benign-appearing” nuclei per epithelial nest, a feature capturing morphologic similarity to non-cancerous tissue (PFI HR=0.48, p=0.001).

The variation between cohorts likely reflects differences in patient populations, variability between pathologists, and limitations of the Nottingham criteria.

### Stromal, immune, and spatial features play an essential prognostic role in breast cancer

**Figure 3:**
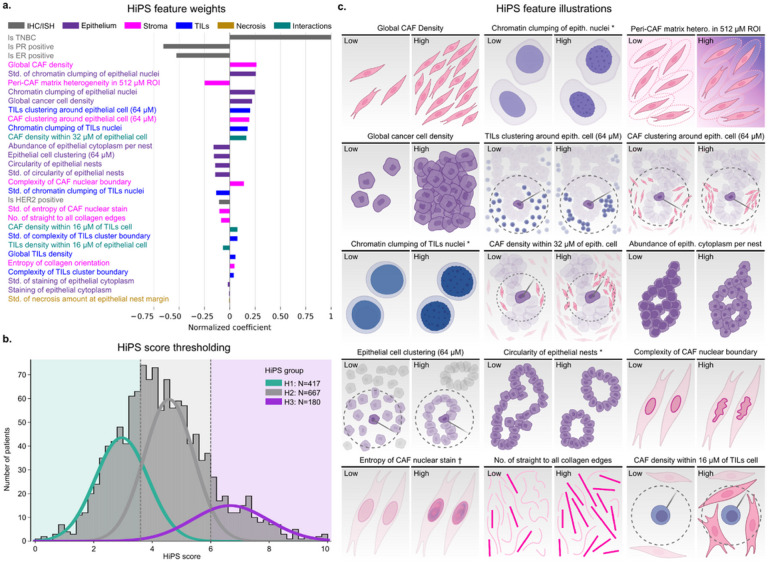
The Histomic Prognostic Signature. a. Relative contribution of histomic features to the HiPS score. HiPS combines 26 computationally-derived morphologic descriptors of the tumor microenvironment from H&E WSI scans with the breast IHC/ISH panel. While epithelial features were influential, we found that stromal, immune, and cell-cell interaction features had an equally important prognostic role. b. Distribution of the HiPS scores among patients from the CPS-II cohort. The distribution was modeled as a mixture of three Gaussians defining low (H1), intermediate (H2), and high (H3) risk prognostic groups. c. Illustrations of the most influential features on the HiPS score, ordered by importance. The star symbol indicates features whose mean and variance values were both influential, while the cross symbol indicates features whose variance alone was influential. Cancer-Associated Fibroblast (CAF) density and acellular stromal matrix heterogeneity were among the top-five features. The morphology and local interactions of CAFs and Tumor-Infiltrating Lymphocytes (TILs) also played an important role. Partially created with BioRender.com.

Our transparent prognostic model reveals the influence of individual features, including (Fig. 3, Table S22):

#### Global CAF and cancer cell densities:

Global density of CAFs was the most important histomic feature and was slightly more influential than cancer cell density. In contrast, global TILs density had little influence.

#### Nuclear chromatin clumping:

The magnitude and cell-to-cell variation in chromatin clumping in cancer cells was highly prognostic, reflecting the nuclear pleomorphism component of Nottingham grading. Surprisingly, chromatin clumping of TILs was also informative.

#### Stromal matrix and collagen fibrils:

The variation in staining of the stromal matrix is the third most influential histomic feature. This reflects interface changes like stromal desmoplasia. We also find the waviness of collagen fibrils to be weakly influential, reflecting general architectural disruption in advanced cancers. In contrast, the entropy of collagen orientation was not influential, likely because its prognostic value is not independent of other features in HiPS.

#### Epithelial-stromal cell interactions:

These include the clustering of TILs and clustering of CAFs within 64 μM of cancer cells, as well as the density of TILs within 32 μM of cancer cells.

#### Epithelial nest morphology and architecture:

These include cell clustering (i.e., formation of nests), nest circularity, and slide-level variation in nest circularity. The amount of cytoplasm per epithelial nest, a robust estimate of the nuclear-to-cytoplasmic ratio, is also influential.

**Figure 4:**
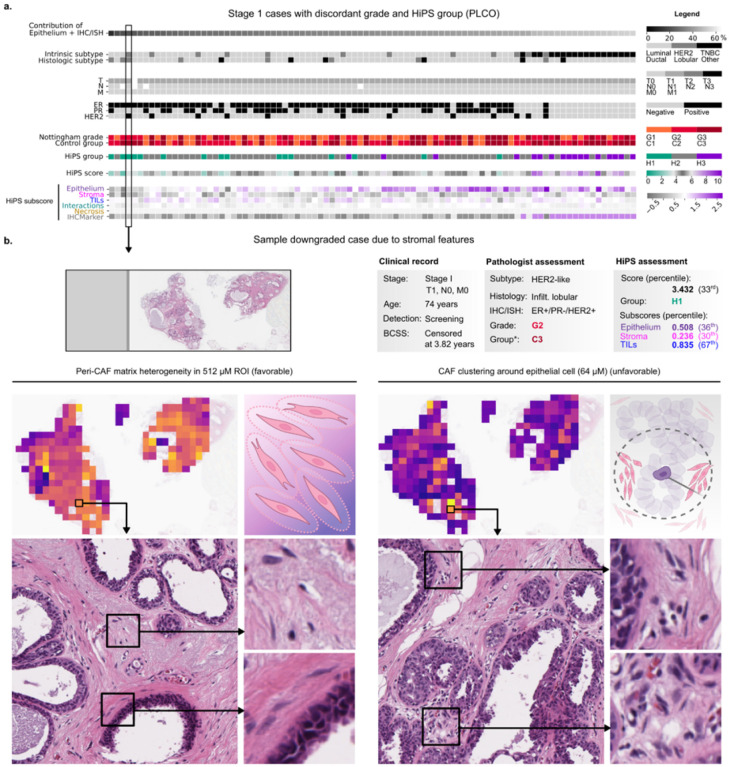
Stromal features critically impact the HiPS score and alter risk categorization in stage I cancers. a.Clinico-pathologic characteristics of stage I cancers where HiPS altered the Nottingham risk categorization (91 of 231 stage I patients). By definition, stage I cancers do not have nodal involvement, so there is a higher importance of histology in guiding clinical decision making. In the supplement we show that HiPS improves outcome stratification in this cohort. Patients are sorted by the percent contribution of epithelial and ER/PR/HER2 features to the HiPS score. We also show the HiPS subscores, each of which summarizes the influence of features within each biological theme. The summation of the six HiPS subscores equals the total HiPS score. Note that non-epithelial features contribute heavily to the total HiPS score in this cohort, and were heavily influential in altering the patients’ risk categories. b. Sample case where stromal features were heavily influential. Two features are illustrated: 1. The variation in peri-CAF stromal matrix intensity, which reflects stromal interface changes like desmoplasia and is favorably prognostic; 2. Clustering of CAFs within a 64 μM radius of epithelial cells, which is adversely prognostic. Partially created with BioRender.com.

The linearity of our prognostic model also allowed us to calculate subscores that summarize the influence of features belonging to a single theme. Hence, the HiPS score is the sum of six thematic subscores (Figs. 4,S30–35). In cases where HiPS altered patients’ risk categorization, we found that the combined contribution of epithelial and ER/PR/HER2 subscores was often less than that of the other subscores. In other words, a patient who harbors high-risk epithelial features may be considered at a lower overall risk because of their prognostically-favorable stromal features, and vice versa. Note that this principle underlies efforts to use stromal/immune biomarkers to guide immunotherapy de-escalation^15,51^.

**Figure 5:**
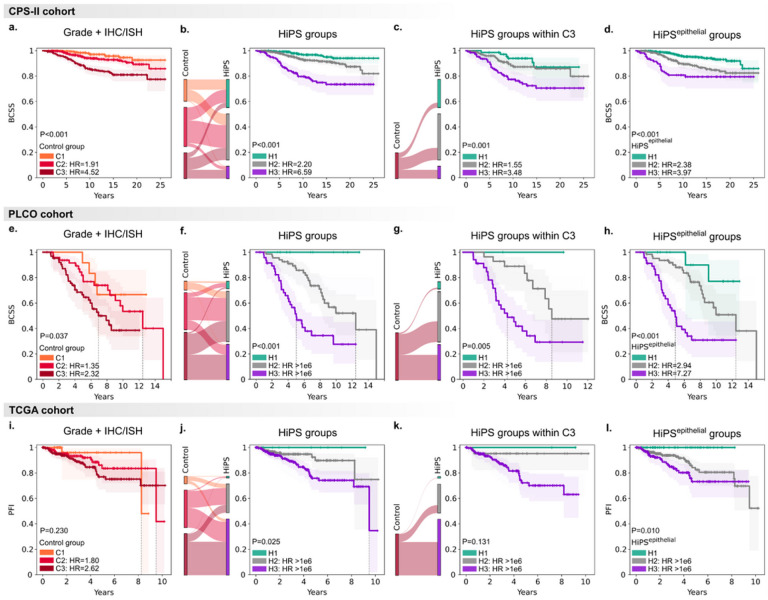
Kaplan-Meier analysis of HiPS groups compared to the Control groups. Detailed results and at-risk tables are provided in Tables S11-16. CPS-II was our discovery cohort, while PLCO and TCGA were independent validation cohorts. Control prognostic groups were obtained by combining Nottingham grades from pathology reports with the ER/PR/HER2 panel using the same methodology as the HiPS score. All P-values represent the Log-Rank statistic, and all hazard ratios (HR) are relative to the lowest risk category. a. Breast cancer-specific survival (BCSS) outcomes for patients within the CPS-II cohort using the Control groups (Nottingham grade + ER/PR/HER2). b. Left: Sankey diagram of reclassification from Control group to HiPS in in CPS-II. Right: BCSS outcomes for patients in the CPS-II cohort using the HiPS groups. Note the higher HR than Control groups in panel a. c. Left: Sankey diagram of reclassification from Control group C3 to HiPS groups within the CPS-II cohort. Right: BCSS outcomes for Control group C3 patients when stratified into HiPS groups. HiPS enables BCSS outcome stratification within Control group C3. d. BCSS outcome stratification for the CPS-II cohort using the alternative model, HiPS^epithelial^, which only relies on epithelial histologic features and the ER/PR/HER2 panel. Group 3 HR values are smaller than those observed with the full HiPS model in panel b. e-h. Equivalent results to panels a-d, but for the PLCO cohort. i-l. Equivalent results to panels a-d, but using Progression-Free Interval (PFI) outcomes in the TCGA cohort. In TCGA, high censorship rates and missing cause of death information make PFI the most suitable outcome measure. Partially created with BioRender.com.

That many stromal and spatial features outweigh epithelial features is consistent with increased recognition of the role of the TME. We hypothesize that HiPS’ prognostic value is partly driven by the diversity of biological phenomena it captures. To test this hypothesis, we performed a sensitivity analysis that fit an alternative score, HiPS^epithelial^, based entirely on epithelial features and the standard IHC/ISH panel (Fig. S8). HiPS^epithelial^ was inferior to the full HiPS score, highlighting the value of fully incorporating TME features. Compared to HiPS^epithelial^, HiPS better stratified BCSS in CPS-II (group 3 HR=6.59 vs. 3.97) and PLCO (group 3 HR=8.3e+7 vs. 7.27) (Fig. 5). HiPS and HiPS^epithelial^ had similar prognostic value in TCGA, which comprises more advanced cancers.

Note that computational assessment of epithelial elements alone is also superior to manual grading; HiPS^epithelial^ improves outcome stratification in all three datasets compared to Control grouping. This is not entirely explained by increased objectivity from computational versus manual grading, since HiPS includes highly prognostic epithelial features not associated with the Nottingham grade (Fig. 1b).

### HiPS is more prognostic than Nottingham grading in independent validation sets

HiPS stratifies patients into three risk groups in CPS-II, PLCO, and TCGA (Figs. 5,S11–18). Within CPS-II, Nottingham grade identifies patients with distinct breast cancer-specific survival (BCSS) (p<0.001). Control groups, incorporating ER/PR/HER2, improve this stratification (C3 HR=4.52 vs. G3 HR=3.99, both p<0.001). HiPS groups are yet more predictive of BCSS (H3 HR=6.59, log-rank p<0.001). HiPS is also a better predictor of overall survival (OS) than Control groups (p<0.001 vs. p=0.065).

A similar result was found in the PLCO and TCGA validation cohorts. In PLCO, HiPS improved BCSS stratification (log-rank p<0.001 vs. p=0.037) and OS stratification (p=0.015 vs. 0.308). We also show that HiPS successfully stratifies OS outcomes in the TCGA dataset, although the result was not statistically significant (p=0.055). We also explored Progression-Free Interval (PFI) and found that HiPS significantly stratified outcomes (p=0.025)^52^. Control groups could not stratify TCGA patients’ OS or PFI outcomes. We hypothesize that overrepresentation of advanced cases from tertiary care centers may drive this failure (TNM Stages 2–3: 82.8% in TCGA vs. 19.6% in CPS-II). TCGA also has fewer observed deaths than CPS-II (9.1% vs. 36.6%, p<0.001).

### HiPS reassigns patient risk categories to be more consistent with observed outcomes

Novel diagnostics are incrementally useful insofar as they reclassify patients into higher or lower risk (Figs. S20–22). To identify clinically-relevant risk category reassignments (i.e., those impacting survival outcomes), we evaluated HiPS within each Control group. In CPS-II, we could identify three prognostically-distinct subsets within C2 (p<0.001) as well as C3 (p=0.001). Likewise, the clinically-relevant reassignments in PLCO were those from C3 to H2, identifying a distinct subset of patients with better survival (median BCSS=8.5 vs. 4.3 years, p=0.005). HiPS also allows some (non-significant) stratification within C3 in TCGA by downgrading a subset of these cases.

### HiPS is prognostic independent of TNM stage and pertinent clinical variables

HiPS score and groups provide significant prognostic value for BCSS in the CPS-II cohort, independent of cancer stage and tumor size (Tables S6–7, Fig. S17) (HiPS score HR=1.28, p<0.001; H3 HR=3.62, p<0.001). The Control score and groups were also independently prognostic (Tables S8–9) (Control score HR=1.24, p<0.001; C3 HR=3.56, p<0.001). When used in an expanded multivariable Cox regression model, HiPS scoring and groups retained their prognostic value (HiPS score HR=1.41, p<0.001; H3 HR=3.45, p=0.006). In addition to tumor stage and size, this model also included demographic factors (age, menopausal status, race, smoking, BMI), Basal marker expression (CK5/6 or EGFR), detection method (screening vs. self-detected), and treatment (chemotherapy, radiation therapy, and targeted therapy) (Tables S10–11). The Control models had similar prognostic value (Tables S12–13) (Control score HR=1.33, p<0.001; C3 HR=3.56, p=0.003).

We also found that HiPS scoring, but not HiPS grouping, was independently predictive of OS within PLCO after adjusting for cancer stage, tumor size, and patient age (Tables S14–15, Fig. S18) (HiPS score HR=1.15, p=0.029). Here we assessed OS rather than BCSS due to missing data and sample size constraints. In contrast, the Control models were not prognostic when adjusted for the same covariates (Tables S16–17).

Likewise, HiPS scoring, but not HiPS grouping, is predictive of OS in the TCGA cohort independent of cancer stage and tumor size (Tables S18–19) (HiPS score HR=1.22, p=0.009). The Control models had no independent prognostic value (Tables S20–21). Neither HiPS nor Control scoring was predictive of PFI.

### HiPS grouping is prognostic within ER+, Luminal-like, and HER2+ breast cancer

HiPS improves outcome stratification compared to grading in ER+, Luminal-like, and HER2+ cancers. As expected, the improvement was more dramatic in the CPS-II discovery cohort compared to the independent cohorts (Figs. S36–41). Although HiPS improves BCSS stratification of ER- cancers in CPS-II, the effect did not generalize to other cohorts. Neither grading nor HiPS could stratify TNBC patients in any cohort.

Within the CPS-II ER+ subcohort, HiPS grouping performs better than Nottingham grading, both in terms of OS (p<0.001 vs. 0.411) and BCSS (H3 HR=6.76 vs. G3 HR=3.83, all p<0.001). We note that Nottingham grades were almost indistinguishable in terms of median OS (20.2-20.5 years), whereas H3 patients had a shorter median OS of 14.9 years compared to 20.6 and 20.2 years of H1 and H2. Similarly, HiPS stratifies BCSS outcomes in the PLCO ER+ subcohort (p=0.004), unlike Nottingham grading (p=0.163). Finally, HiPS results in improved, albeit not statistically significant, stratification of OS and PFI outcomes of the TCGA ER+ subcohort.

Because most luminal-like cancers are ER+, the prognostic effect of HiPS is similar in these subgroups. In CPS-II Luminals, HiPS results insignificant stratification of OS and BCSS outcomes compared to Nottingham grading (OS: HiPS p=0.001, vs. Nottingham p=0.565; BCSS: HiPS p<0.001, G3 HR=6.73, vs. Nottingham p<0.001, G3 HR=4.33). In TCGA Luminals, HiPS grouping results in a visible improvement in OS and PFI stratification but is not statistically significant. In contrast, neither Nottingham nor HiPS could significantly stratify outcomes in the PLCO Luminal-like cohort.

Finally, HiPS grouping of CPS-II HER2+ cancers result in a dramatic improvement in the stratification of outcomes compared to grading (OS: p=0.035 vs. 0.561; BCSS: p=0.005 vs. 0.541). Likewise, HiPS improves OS stratification in HER2+ cancer in PLCO (p=0.051 vs. 0.690). Missing cause of death data precluded BCSS assessment in this subcohort. Neither Nottingham nor HiPS grouping could significantly stratify the HER2+ TCGA subcohort.

### The HiPS score is consistent with established survival risk profiles

**Figure 6:**
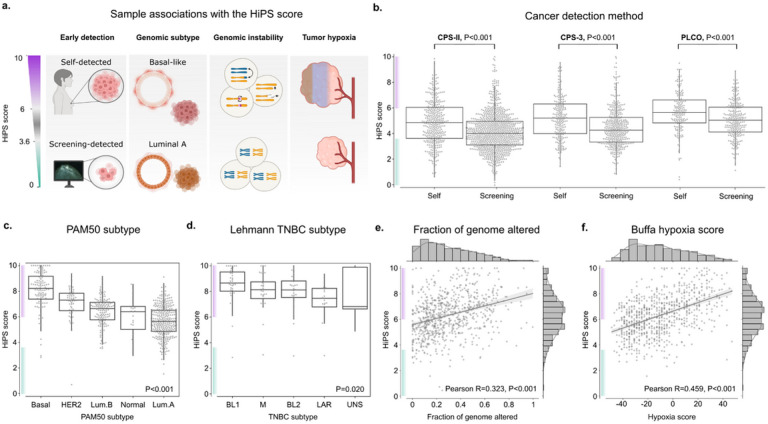
The HiPS score is consistent with established risk profiles. In each of the plots shown, the green and purple y-axis ranges indicate H1 (scores <3.6) and H3 (scores ≥6), respectively. Supporting results and sample sizes are provided in Tables S1-2,S27. P-values for panel b represent the independent two-sample t-test, while those in panels c-d represent the one-way ANOVA. a. Illustrating some of the epidemiological and genomic associations discussed. b. The distribution of HiPS scores by cancer detection method. Cancers detected using screening programs had lower HiPS scores than self-detected ones, likely reflecting early detection before developing high-risk features. c. HiPS score distributions within the PAM50 genomic subtypes. Basal and Luminal A cancers have the highest and lowest HiPS scores, consistent with known risk profiles. d. HiPS score distributions within genomic TNBC subtypes. Consistent with known mutational burdens, Basal-Like 1 (BL1) cancers have the highest scores, while Luminal Androgen Receptor (LAR) cancers have the lowest scores. UNS represents an unspecified TNBC subtype. e-f. Scatter plots of HiPS scores versus the fraction of genome altered and the composite Buffa hypoxia score. Higher genome alteration and tumor hypoxia correlate directly with the HiPS score. Partially created with BioRender.com.

The HiPS score is higher in cancers with high-risk clinical and genomic features (Fig. 6). Cancers that were detected using mammographic screening had lower HiPS scores than those self-detected in the CPS-II, CPS-3 and PLCO cohorts (all P<0.001). This finding is consistent with recommendations for cancer screening by the ACS. This association persisted within TNM stage I (all P≤0.001) and stages II-III (P=0.002, 0.014, <0.001 in CPS-II, CPS-3, and PLCO). Screening-detected cancers derived their low-risk scores not only from their ER/PR/HER2 status but because they also had favorable histology. This was evidenced by the lower HiPS scores in screening-detected cancers within luminal-like cancers (all P≤0.001), HER2-like cancers (P<0.001 for CPS-II, CPS-3. PLCO not significant), and TNBC (P=0.037, 0.006 for CPS-II, PLCO. CPS-3 not significant).

PAM50 gene expression subtypes had significantly different HiPS scores in TCGA (p<0.001), with Basal and Luminal A cancers having the highest and lowest scores, respectively (8.05±1.46 vs. 5.64±1.23). Likewise, higher HiPS scores are observed in cancers expressing the Basal IHC/ISH markers EGFR (in CPS-II and CPS-3, p<0.001) and CK5/6 (in CPS-II, p<0.001). In TCGA, the HiPS score was significantly correlated with composite scores measuring the accumulation of genetic alterations, including the Aneuploidy Score (r=0.228, p<0.001) and the fraction of genome altered (r=0.323, p<0.001)^53,54^. It was also correlated with various composite measurements of tumor hypoxia, including the Buffa, Ragnum, and Winter Hypoxia scores (r=0.355–0.459, all p<0.001)^55–58^.

Within TCGA triple-negative breast cancers (TNBC), HiPS score distributions differed by genomic subtype (p=0.02), with Basal-Like 1 (BL1) and Luminal Androgen Receptor (LAR) having the highest and lowest scores, respectively (8.53±1.36 vs. 7.30±1.37). This is consistent with the mutational burden in BL1 cancers compared to LAR (2.1 vs. 1.8 mutations/Mb)^59^. HiPS scores were inversely correlated with immune cell infiltration within the TNBC microenvironment, determined using the xCell genomic deconvolution assay (r=-0.179, p=0.039) or the ESTIMATE immune score (r=-0.168, p=0.05)^60,61^.

## Discussion

We described the development, validation, and utilization of a computational histologic signature for prognosticating invasive non-metastatic breast cancer using population-based datasets. HiPS is a multimodal score that combines scanned H&E slides with the ER/PR/HER2 panel to place patients along a risk spectrum based on the prognostic favorability of their TME. Our method combines deep learning for robust panoptic segmentation of tissue regions and cell nuclei, morphological processing to extract hypothesis-driven features, and Cox regression modeling to maximize interpretability. This approach enabled us to capitalize on recent advances in deep learning for pattern recognition while addressing interpretability issues that limit widespread adoption of deep learning in clinical settings. We developed and validated our score using two population-level cohorts from the CPS-II and CPS-3 studies and two diverse datasets from the TCGA and PLCO trial. In total, we used data from 3,177 patients, with tissue samples from 614 counties in 48 states. These data have a high variability in patient demographics, slide preparation and staining protocols, WSI scanners, and other preanalytical factors. This diversity supports the generalizability of our findings.

Quantitative HiPS features were developed in a hypothesis-driven manner to quantify distinct biological phenomena^62,63^. As a result, we exceeded expert performance using established grading criteria. This success is partly driven by capturing stromal, immune, and spatial clustering features not typically assessed. However, we exceeded human performance even when we limited our analysis to epithelial morphology. These gains may be due to the quantitative nature of HiPS compared to visual estimates^64^. Nonetheless, we also showed that epithelial features modestly correlated with the Nottingham criteria are highly prognostic, consistent with prior indirect evidence^23^.

Compared to prior works, our segmentation models enable us to untangle the influence of CAFs, acellular stroma, and TILs^65,66^. In particular, we found that stromal interface changes reflecting desmoplasia or collagen disorder were favorably prognostic, consistent with prior works^65,67^. We also found an adverse prognostic value of CAF density, both globally and within 64 *μ*M of epithelial cells, which may be proxies for wound healing and epithelial-CAF interaction^13,68^. Also, consistent with Yuan *et al*., we found CAF clustering around epithelial cells to be adversely prognostic^69^. Some of our measurements also focused on phenotypic differences in the appearance of CAFs. We found that increased average complexity of CAF nuclear boundary is adversely prognostic, possibly reflecting myoepithelial differentiation or epithelial-to-mesenchymal transition (EMT) of leading cancer cells as they acquire a CAF-like morphology^13,70^.

We found TILs clustering and morphology to be more relevant than their abundance. This may reflect the inconsistent prognostic nature of TILs abundance in ER+ (unfavorable) versus TNBC and HER2+ cancers (favorable)^71,72^. In particular, the spatial clustering of TILs within 64 *μ*M of epithelial cells is highly prognostic, likely reflecting TIL-TIL interactions that fuel the inflammatory response sustaining or modulating cancer progression^73,74^. Moreover, we found that the morphology of TILs nuclei is prognostic. High average nuclear chromatin clumping is an adverse prognostic biomarker, while the TlL-to-TIL variation in chromatin clumping is favorable. The biological significance of these findings is unclear but could represent different lymphoycte subsets and degrees of differentiation^75^. Future work using IHC/ISH-based analysis would address this; we focused on H&E to ensure applicability in routine settings^76^.

We want to highlight some of the limitations of our approach. The prognostic value of histomic features is dependent on multiple factors, not just fundamental biology. For example, the robustness of algorithms in consistently capturing the same phenomena is an important consideration. We described each tissue region and cell nucleus by a set of morphological and spatial features, which were aggregated using weighted mean and variance to obtain per-patient results. This results in the loss of potentially useful information, such as the outsized influence of small foci of angioinvasion. However, there is an inevitable tradeoff between modeling complexity and interpretability^43,45^. Our results show that HiPS is not prognostic within ER- and TNBC cancers, which may reflect a differential effect of histomic features depending on intrinsic subtype. We intend to explore this in future works. Finally, we acknowledge the observational nature of this analysis. Most of the histomic features have correlates in tumor biology, but the causative relationship is unclear.

## Online Methods

### Clinical datasets

TCGA clinical and WSI data was obtained from the National Cancer Institute Genomic Data Commons (GDC) portal: https://gdc.cancer.gov/. Updated survival outcomes data were obtained from the supplemental files from Liu *et al*.^52^. Breast cancer genomic subtype classification, precalculated Buffa/Winter/Ragnum hypoxia scores, fraction of genome altered, and aneuploidy scores were all obtained from the PanCancer Atlas on GDC: https://gdc.cancer.gov/about-data/publications/pancanatlas^53,54^.

The CPS studies are prospective observational cohort studies of cancer risk factors, morbidity, and mortality that the American Cancer Society conducted. Deidentified clinical and imaging data was shared with Emory University and Northwestern University through data sharing agreements. Adult men and women with no personal history of cancer were enrolled in 1982 in CPS-II and 2006-2013 in CPS-3^27,77,78^. CPS-II participants enrolled in a subcohort followed for cancer incidence and mortality starting in 1992/1993^24^. CPS-II cancer incidence follow-up is complete through 2017 (mortality through 12/31/2018). In CPS-3, follow-up is ongoing but currently complete through 2018 (mortality through 2017). We included female participants who developed breast cancer and for whom tissue slides were available for scanning. The included participants were diagnosed between the start of follow-up (CPS-II: 1992–1993, CPS-3: 2006–2013) and the last administrative date (CPS-II: 2017, CPS-3: 2018).

The Prostate, Lung, Colorectal, and Ovarian (PLCO) Cancer Screening Trial was designed to evaluate the efficacy of specific screening procedures in reducing mortality rates associated with prostate, lung, colorectal, and ovarian cancers^26^. This PLCO trial adopted a randomized, controlled design and recruited participants from 1993 to 2001. Cancer incidence data were amassed until December 31, 2009, while mortality information was collected through 2015. PLCO female participants who developed breast cancer after enrollment in the trial were included in our study. Deidentified clinical and WSI data were obtained with permission from the National Cancer Institute Cancer Data Access System.

### Geographic data processing

United States state and county data presented in Fig. 1 correspond to facilities where the tissue samples were sourced. Cartographic boundary data was obtained from census.gov for 2021: https://www.census.gov/geographies/mapping-files/time-series/geo/cartographic-boundary.html. Facilities in cities that intersect multiple counties were assigned the county that maximally intersects the city, as determined using: https://simplemaps.com/data/us-cities. Facilities in small towns were assigned to the nearest city.

### WSI data acquisition and management

CPS-II and CPS-3 slides were scanned at Emory university using the NanoZoomer 2.0-HT slide scanner by Hamamatsu Photonics at a 40x magnification (0.23 μm/pixel). They were saved as *.ndpi* files. PLCO WSIs were obtained utilizing the Aperio AT2 (Leica) scanner at a 40x magnification using standard settings. They were saved as *.svs* files at the Cancer Genomics Research Laboratory at the National Cancer Institute. TCGA slides were scanned at different institutions that contributed samples to the data repository; details can be found at the Genomic Data Commons Portal (https://portal.gdc.cancer.gov/), and a detailed analysis of site-specific characteristics was provided by Howard *et al*.^79^.

All WSI management was done using the Digital Slide Archive software platform: https://digitalslidearchive.github.io/digital_slide_archive/^50^. This platform includes a Mongo database that is accessible through a user interface as well as programmatically through RestfulAPI. Additionally, we utilized the associated image processing library (HistomicsTK) and WSI visualization and annotation interface (HistomicsUI).

### Programming and statistical analysis

All computer programming for clinical and WSI data analysis was done using the Python and Bash programming languages. Deep learning models were developed using the Pytorch library. Statistical tests for specific experiments were discussed with relevant figure and table captions.

Unless stated otherwise, all statistical tests were two-sided, and all measurements were taken from distinct patient samples. Pearson and Spearman correlations, independent-sample t-test, and the one-way ANOVA statistics were calcluated using the *pearsonr, spearmanr, ttest_ind*, and *f_oneway* methods of the *scipy.stats* library using default settings. The *lifelines* python package was used for survival analysis, including the *KaplanMeierFitter* and *CoxPHFitter*, and *multivariate_logrank_test* using default parameters. For all boxplots shown, graphical elements represent the standard representation: center line, median; box limits, upper and lower quartiles; whiskers, 1.5x interquartile range; points, outliers.

### Illustrations

Multiple figure panels were created with BioRender.com. Most plots were created using the *seaborn* and *matplotlib Python* libraries and compiled using *Inkscape*.

### Panoptic segmentation model training

We used our panoptic segmentation Convolutional Neural Network (CNN) model, *MuTILs*, to delineate tissue regions and cell nuclei in the slide, a task we hereafter refer to as *panoptic segmentation* given that it combines semantic segmentation (regions) and object detection (nuclei) (Figs. 1b)^46,47^. Figs S2–5 illustrate MuTILs segmentation results on five representative WSIs from the PLCO dataset. We had previously published details of the training and validation procedure, but we summarize it below for convenience^46^.

The MuTILs architecture is composed of two U-Net CNN models that work in parallel to segment regions and nuclei at resolutions of 1 and 0.5 microns-per-pixel (MPP), respectively^80^. It is a multi-resolution, multi-task, biologically inspired CNN architecture. To transfer information from the low-resolution branch to the high-resolution branch, we utilized concatenation, inspired by the HookNet architecture^81^. Region predictions were used to impose constraints on the nucleus class inference to ensure compatibility of the region-level and cell-level predictions. This was achieved using class-specific attention maps, which were derived by modeling the nucleus class prior probability as a linear combination of the corresponding region probability vector. We trained MuTILs on WSI data from two publicly available datasets: the Breast Cancer Semantic Segmentation dataset (region segmentation) and the NuCLS dataset (nucleus detection)^48,49^. Region-level and nucleus-level data were reconciled to produce a single panoptic segmentation dataset^46^. Additionally, we expanded the *training* data by annotating 85 slides from the CPS-II cohort to improve representation of less-advanced cases.

To evaluate our model’s performance, we partitioned the slides using 5-fold internal-external cross-validation; i.e., different institutions were assigned different training/testing folds. All testing set annotations were produced or reviewed by pathologists, as described in prior work^48,49^. MuTILs accuracy statistics include :

- Cancer region segmentation: DICE= 82.7 ±0.4
- Stromal region segmentation: DICE= 80.8 ±0.4
- Cancer nucleus detection: AUROC= 95.9 ±3.2
- CAF nucleus detection: AUROC=91.0 ±3.6
- Lymphocyte nucleus detection: 93.0±1.1
- Nucleus detection Micro-average: 92.6±2.8
- Nucleus detection Macro-average: 82.7±5.0

Other forms of validation were used as well, including qualitative examination of inference results and correlation between computational and manual TILs scores (Spearman R=0.58, P<0.001). Please refer to our prior work for further details^46^.

### Panoptic segmentation of WSIs

After we had trained the MuTILs model, we had five sets of weights, each trained on a different fold of training data obtained from a unique set of hospitals (internal-external cross-validation)^82^. We relied on this to reduce the overall bias in our predictions by employing a form of model ensembling that does not increase overall run time and hence more closely reflects realistic clinical deployment. We obtained the region-adjacency graph for each WSI at a very low resolution (20 MPP). Using the *rag_threshold* function in the *histolab* library, the low-resolution image was segmented into superpixels using the Simple Linear Iterative Clustering (SLIC) algorithm, which were then clustered using K-means based on the intensity values^83,84^. We set the SLIC parameters to 128 segments, a compactness of 10, and a threshold of 9. Each contiguous segment was assigned a single set of MuTILs model weights. In effect, every low-resolution superpixel was predicted by one set of weights, and each set of model weights could be assigned multiple superpixels **(Fig. S6)**. Using this approach minimized edge artifacts related to systemic biases in predictions from different model weights.

First, we excluded white space and red/green/blue markings (felt-pen or inking) using the relevant *histolab* functions, which rely on color thresholding. Next, we tiled each WSI into 512×512 μM square ROIs, keeping only tiles with at least 50% tissue composition. Second, we performed deconvolution-based color normalization using the Macenko method, restricting the color normalization to the tissue mask using the *HistomicsTK* library^85^. Third, each ROI was assigned a single set of MuTILs model weights for inference depending on which superpixel it maximally overlaps with. Once we had obtained the CNN inference results for regions and cells, we saved a WSI segmentation mask for later use in feature extraction.

### Histomic feature extraction

Using our previously trained and validated panoptic segmentation model (MuTILs), we were able to delineate all tissue regions and nuclei within WSIs automatically. These regions and nuclei were then used to extract several morphological and contextual features. Because each slide contains thousands of regions and up to a million nuclei, these features were aggregated to obtain a single mean and variance value per patient, as we discuss in the next section. After feature aggregation, each patient sample was thus described by a set of 109 numbers, i.e., the patient-level histomic features. Table S5 describes each of those features, and we provide a general overview below:

#### - Global features:

Such as overall cancer cell density, overall TILs density, the global amount of necrosis within the WSI, etc. These values were normalized to the amount of tissue analyzed.

#### - Region morphology:

Standard size and shape measurements were extracted for each tissue region, focusing on epithelial and TILS-dense regions. While we segment tissue regions at a 1 MPP resolution. The output is saved as a WSI mask, which is then loaded for region feature extraction at a 2 MPP resolution. Saving the WSI mask enabled us to extract features from contiguous tissue regions that may span multiple ROIs; hence this approach mitigates the effects of artifacts related to the ROI edge. To obtain individual regions from the semantic segmentation mask, we first did binary dilation of the mask using a 5×5 pixel selem (i.e., 10×10 μM) to remove small artifacts. Then, we removed holes smaller than 48^2^ μM^2^in area. Finally, we performed a connected component analysis using a connectivity of 2. Only tissue regions with an area larger than 128^2^ μM^2^ were considered.

Region morphology features were extracted using the *sklearn* library’s *regionprops* method. Boundary complexity was measured using fractal (box counting) dimension, as implemented by Nicholas P. Rougier in this GitHub repository: https://gist.github.com/rougier/e5eafc276a4e54f516ed5559df4242c0. We note that the MuTILs model detects contiguous TILs aggregates at one MPP resolution. Hence, we did not need to use global clustering or graph-based methods to identify TILs cluster boundaries as they were determined in a data-driven manner using semantic segmentation.

#### - Standard nuclear morphology:

Nuclear features were extracted for the three nuclear superclasses: epithelial, non-TIL stromal (mostly CAFs), and TILs. Nuclear size, shape, staining intensity, boundary complexity, edges (chromatin clumping), and texture were extracted using the *HistomicsTK* library’s *compute_nuclei_features* method: https://github.com/DigitalSlideArchive/HistomicsTK. The HistomicsTK implementation is primarily based on the *sklearn* library’s *regionprops* method for the size and shape features, Canny edge detectors and Haralick features for texture, and fractal dimension for boundary complexity^28^.

#### - Deep nuclear morphology:

We observed that nuclei do not always have typical morphology and that they are often ambiguous and difficult to classify in H&E images without IHC markers. Deep-learning models produce a classification probability vector, and we used those probabilities to capture the degree of conformity of nuclei to various classifications of interest.

#### - TILs activation:

The probability that a particular nucleus is classified as a plasma cell, divided by the overall probability that the nucleus belongs to the TILs superclass. In the ground truth, the plasma cell class was not determined using IHC, nor was it limited to the most typical morphology. Hence, it refers to large TILs, including plasma cells and others.

#### - Epithelial nuclear atypia:

The probability that a certain nucleus is classified as a cancer cell divided by the total probability that it belongs to the epithelial class.

#### - CAF epithelialization:

The probability that a nucleus is classified as a fibroblast, divided by the sum of its fibroblast and cancer cell classification probabilities.

#### - Cytoplasmic texture and staining:

The delineation of cytoplasmic boundaries in H&E is unreliable, so we calculated texture statistics within 4 μM of nuclear boundaries. This search area is determined by dilating nuclear boundaries. The *HistomicsTK* library was used for extracting these features.

#### - Local cell density:

These were defined as the average number of cells of a certain class within a predefined radius from the “central” cell. The central nucleus can have the same class as the surrounding nuclei; for example, the *LocalTILsDensity32uM* metric measures how many TILs are within 32 μM of the typical TILs cell. Alternatively, the central and surrounding can have different classifications; for example, *TILsDensityWithin32uMOfEpithCell* measures how many TILs are within 32 μM of the typical epithelial cell. This statistic was calculated using a fast K-D trees implementation, loosely based on the implementation by Sam P. Ingram in this Github repository: https://github.com/SamPIngram/RipleyK.

#### - Local cell clustering:

These were based on Ripley’s K-function at a single distance, which is a measure of clustering beyond that expected from random chance^86,87^. We obtained this metric by normalizing local cell density estimates to “Complete Spatial Randomness.” For example, there is a higher chance that more lymphocytes will surround another lymphocyte by random chance, just because there are so many of them. Hence, high density does not necessarily indicate clustering beyond random chance. On the other hand, just a few fibroblasts surrounding each other may result in a high clustering value since they are (globally) less dense, so there is a lower chance of this dense local aggregation occurring by random chance. The radii used for the calculation of local cell density and clustering were 16, 32, and 64 μM.

#### - Region composition:

Cellular composition of various histopathological tissue compartments. For example, *NoOfLowGradeNucleiPerEpithNest* measures the number of epithelial nuclei that were considered low-grade by the MuTILs model, per epithelial nest. Region composition metrics also enabled us to estimate the nuclear-to-cytoplasmic (N/C) ratio. Again, cytoplasmic boundaries cannot be precisely determined in H&E slides, so we relied on the following heuristic to calculate the N/C ratio: divide the total nuclear area within an epithelial nest by the overall area of the epithelial nest.

#### - Region neighborhood composition:

Region masks were morphologically dilated to identify the tissue and cellular composition within 128 μM and 256 μM of the edge. For example, *CAFDensityAtEpithNestMargin* measures the density of CAFs within 128 μM of epithelial nests.

#### - Acellular stromal matrix and collagen:

**Fig. S10** illustrates features that capture stromal matrix, including abstract texture/intensity measurements, as well as a more sophisticated analysis of the separation, length, and disorder of collagen fiber orientations. We captured collagen disorder by three separate approaches.

First, we hypothesized that collagen separation and stromal matrix discoloration (e.g., desmoplasia) would reflect on abstract intensity and texture measurements from the collagen stroma. *PeriCAFMatrixHeteroIn512uMROI* is a feature that captures the variation in stromal matrix at the interface between desmoplastic and quiescent stroma. The metric is calculated by measuring the average intensity within a very thin rim around each fibroblast and calculating the variance in that intensity across a 512×512 μM squared region of interest (ROI). This metric is related to one of the prognostic stromal features described by Beck *et al*., who relied on the absolute difference in intensity between neighboring contiguous stromal regions^65^. Their approach may have been liable to some confounding by segmentation errors or non-stromal matrix elements like small vessels and vacuoles. To address this issue, we relied on the peri-fibroblast stromal matrix within 4 μM. All images were color normalized using the Macenko method to maximize robustness to staining and scanner differences^85^.

Second, we took a direct approach whereby we detected the collagen fibers themselves, largely following the methodology described by Li *et al*.^67^. This analysis used 256×256 μM ROIs with at least 30% stroma and 20% tumor at a 0.5 MMP resolution. We used a Canny edge detection algorithm to detect the interface where collagen fibers separate. Then we used connected component analysis to isolate individual edges. Fibers with a minor-to-major axis ratio <0.2 were considered straight fibers and were further admitted to calculate the CFOD metric described by Li *et al*.^67^. This measures the degree of disorder in collagen orientation, calculated from a length-weighted orientation co-occurrence matrix. One difference between our implementations is that we masked out nuclei before applying the edge detection in order to minimize confounding by nuclear material.

Finally, we also measured collagen entropy indirectly by calculating the entropy of orientations of fibroblast nuclei within a certain radius of each other. We hypothesized that in some settings, fibroblast nuclei might be more reliably detected than collagen fibers.

### Histomic feature aggregation and processing

Histomic features had to be aggregated to obtain patient-level data from tissue region-level, cell-level, ROI-level, and slide-level data. For each patient, we had 1000+ tissue regions, 100,000+ nuclei, multiple ROI-level histomic features (including descriptors of the acellular stroma), and 1–3 slides. Fig. S9 illustrates the ROI-level spatial variability in two influential features before WSI-level aggregation: *ChromatinClumpingOfEpithNuclei* and *PeriCAFMatrixHeteroIn512uMROI*. **Table S5** provides details of the various levels of aggregation used for different features, and we provide an overview below:

- Individual tissue region morphology and neighborhood information were aggregated to obtain per-WSI mean and standard deviation.
- Nuclear clustering, interaction features, and global densities were obtained for the entire WSI to avoid artifacts related to artificial ROI boundaries. No aggregation was needed.
- Individual nuclear morphology was first aggregated per 512×512 μM ROI using mean and standard deviation. Each ROI was assigned a *saliency score*, which is maximized for ROIs with a high composition of adjacent epithelial and stromal tissue regions, hence prioritizing the cancer-stroma interface^46^. The saliency score is calculated as follows:
Saliency=(Areaofstromawithin32μMofanepithelialnest×Areaofepithelialtissue)/(Areaofnon-necrotictissue).

Note that solely relying on stroma within *32 μM* from the tumor is inadequate, as ROIs with scattered, spaced-out tumor nests would get a high score even though there’s little tumor. The per-ROI data were aggregated using saliency-weighted mean and standard deviation, restricted to the top 128 most-salient ROIs.

- Features describing the acellular stromal matrix and collagen were aggregated per-WSI using saliency-weighted mean and standard deviation of the per-ROI data, restricted to the top 256 most-salient 256×256 μM ROIs. Saliency was calculated in the same way as previously described.
- Finally, per-WSI data from multiple slides were averaged to obtain per-patient information.

After aggregation, all histomic features were z-scored relative to the CPS-II mean, omitting missing values. When features had missing values, this was due to unavailable tissue, such as when there were no TILs clusters to calculate cluster morphology. These values were filled using 10-nearest neighbors imputation using the CPS-II cohort.

### Thematic classification of histomic features

Histomic features fall under five major themes, which are further divided into 26 sub-themes. Themes and sub-themes were engineered in a hypothesis-driven manner and are meant to capture distinct biological phenomena and processes (Fig. 2). The themes are epithelial features, stromal features, TILs features, necrosis abundance, and features capturing various interactions between different cells. Sub-themes encompass specific phenomena. For example, the TILs theme is subdivided into TILs abundance, TILs clustering, TILs cluster morphology, TILs cluster pleomorphism, and so on. We expect histomic features within the same themes and sub-themes to be correlated with each other and less correlated with other themes. The exception, of course, is the Interactions theme, which may or may not be correlated with others. Examining the absolute correlation matrix, we can see that the correlation is stronger towards the diagonal, within the squares representing themes and sub-themes (Fig. 2). One of the themes we focused on is characterizing cancer-associated stroma since standard Nottingham grading does not capture it. Stromal features include morphological descriptors of fibroblast nuclei, characterization of TILs, and detailed analysis of the stromal matrix, which is primarily composed of type I Collagen fibers (Fig. S10). All of these measurements were assessed as potential prognostic indicators, and the most prognostic ones were admitted into the final prognostic score.

### HiPS prognostic model fitting

The most prognostic feature from each biological sub-theme at the univariable level was admitted into this model, along with the standard IHC marker panel: Estrogen Receptor (ER) expression, Progesterone Receptor (PR) expression, and HER2+ overexpression (Fig. 2). Additionally, we created a composite metric for triple-negative status (TNBC), defined as the absence of all ER/PR/HER2 markers. The rationale for incorporating IHC markers is to find histomic features that provide excess prognostic values beyond that already defined by the expression of hormone and HER2 receptors. We did not want to learn histological features that were highly correlated with, say, TNBC status since they would not be clinically helpful. In a clinical setting, the practicing pathologist and oncologist always have access to at least the histological slide and the ER/PR/HER2 IHC/ISH markers^3,88^.

A total of 30 features (26 sub-themes and 4 IHC panel features) were entered into an elastic-net regularized Cox proportional hazards survival model, predicting breast cancer-specific survival with the CPS-II patient cohort^89^. The optimal hyperparameters (alpha and L1 ratio) for model regularization were obtained by cross-validation **(Fig. S7,S42)**. The trained model was then used to predict the log partial hazard for the entire training population, and the predictions were scaled such that the resultant continuous score ranges from 0 (lowest risk) to 10 (highest risk). Additionally, we modeled the predicted risk scores as a mixture of three Gaussian curves, representing the low-, intermediate- and high-risk populations. The points where curves cross were then considered a data-driven cut-off for dividing patients into three survival risk groups.

To ensure a fair head-to-head comparison of our Histomic Prognostic Score\, we used the same methodology to develop a baseline model fit only to the Nottingham grade and the standard ER/PER/HER2 panel **(Fig. S7)**. This baseline model also yields a risk score in the range 0-10 and three risk groups. Since grading is discrete, unlike our histomic features, the resulting histogram of predicted risks contained discrete bins, so a mixture of Gaussian could not faithfully represent it. Instead, we divided the score range into three equal intervals.

### Multivariable Cox Proportional Hazards regression

After we had learned the optimum combination of histomic features comprising the HiPS and Control models, and the optimum thresholds to learn discrete risk groups, we produced the following features for each patient:

- *Histomic Prognostic Score (HiPS)*: This number ranges from 0–10.
- *Histomic Prognostic Group*: One of three risk groups: H1, H2, and H3, corresponding to low, intermediate, and high-risk.
- *Control Score*: Ranges from 0–10, using the baseline model (Nottingham grade and ER/PR/HER2).
- *Control Group*: One of three risk groups, C1, C2, and C3, corresponding to low, intermediate, and high-risk.

Using the CPS-II cohort, we fit multivariable models to predict Breast Cancer-Specific Survival (BCSS) using each of the risk scores/groups. There was missing clinical data, so we explored two multivariable models. The first only controls for pathologic stage and tumor size and is a robust model with maximal sample size. We also fit another model using a smaller set of patients with complete clinical information on pathologic TNM stage, tumor size, whether the cancer was detected using proactive screening, menopausal status at diagnosis, race, smoking history, age at diagnosis, body-mass index, and expression of basal markers CK5/6 or EGFR.

We explored the independent prognostic value of the risk scores/groups in the PLCO and TCGA datasets as well. However, we were limited by the constraints of missing data limiting the power of prognostic modeling. The clinico-epidemiologic variables for which data were missing differed for different patients; for example, we may have the cause of death information but not the race. Hence, we used OS in PLCO and TCGA and always controlled for TNM stage and tumor size. Guided by model fit statistics (primarily the concordance index), we also controlled for patient age in PLCO and gene expression intrinsic subtype in TCGA.

We checked hazard proportionality using the *check_assumption* function of the *lifelines* package using a p-value threshold of 0.01: https://lifelines.readthedocs.io/en/latest/jupyter_notebooks/Proportional%20hazard%20assumption.html. We also visually inspected the scaled Schoenfeld residuals as needed. Depending on context, we prioritized answering the preset hypotheses over post-hoc changes to model design^90,91^.

## Data Availability

**Table S27** contains our calculated histomic feature values, HiPS scores and subscores, and related data for the TCGA cohort. We provide this to facilitate reproducibility and to act as a resource for the scientific community. TCGA clinical data and WSIs are publicly available at: gdc.cancer.gov. The Breast Cancer Semantic Segmentation dataset is available at: github.com/PathologyDataScience/BCSS, while the NuCLS dataset is available at: sites.google.com/view/nucls. These datasets were combined to produce the PanopTILs dataset, available at: sites.google.com/view/panoptils. Requests for CPS-II or CPS-3 data should be submitted to the ACS. Requests PLCO data should be submitted at: cdas.cancer.gov/learn/plco.

## Abbreviations

ACS: American Cancer Society
Coef.: coefficient
BCSS: breast cancer-specific survival
ER: estrogen receptor
HER2: Human EGFR receptor 2
HiPS: Histomic Prognostic Signature
HR: hazard ratio
HRT: hormone replacement therapy
IHC: immunohistochemistry
MPP: microns per pixel
OS: overall survival
PFI: progression-free interval
PR: progesterone receptor.
TILs: tumor-infiltrating lymphocytes
TNBC: triple-negative breast cancer
WSI: whole-slide image
